# The stress distribution of a primary molar tooth restored with stainless steel crown using different luting cements

**DOI:** 10.1186/s12903-024-04038-7

**Published:** 2024-02-23

**Authors:** Mehmet Sami Guler, Cigdem Guler, Nihal Belduz Kara, Didem Odabasi, Muhammed Latif Bekci

**Affiliations:** 1 Department of Machinery and Metal Technologies, Vocational School of Technical Sciences, Ordu University, Ordu, Turkey; 2 Department of Pediatric Dentistry, Faculty of Dentistry, Ordu University, Ordu, Turkey

**Keywords:** Finite element analysis, Stainless steel crown, Resin cement, Glass ionomer cement, Primary teeth

## Abstract

**Background:**

The aim of this study is to evaluate the stress distributions of a primary molar tooth restored with a stainless steel crown (SSC) using resin and glass ionomer luting cements by Finite Element Analysis (FEA).

**Methods:**

Original DICOM data of a primary molar was used to create a 3D model. One model was prepared as a tooth model with SSC. A 30 μm cement layer was used in model. Two different luting cements were tested in the study: self-cure adhesive resin cement, and glass ionomer cement. Vertical and oblique loads of 330 N were applied to simulate maximum bite force and lateral forces in the occlusal contact areas of the models. Maximum von Mises stress values in the models were evaluated as MPa.

**Results:**

The maximum von Mises stress value was observed in the force application and general occlusal contact areas for all models. The maximum von Mises stress values were higher in the tooth model with SSC using self-cure adhesive resin cement (478.09 MPa and 214.62 MPa) than in the tooth model with SSC using glass ionomer cement (220.06 MPa and 198.72 MPa) in both vertical and oblique loading, respectively.

**Conclusions:**

Depending on the magnitude of the bite force on the SSC, fracture of the luting cement materials could occur if the stress exceeds the endurance limit of the luting cement. Cementation with glass ionomer cement may help to reduce stress levels in SSC restorations of primary molars in children.

## Background

For many years, dentists have been working to restore excessive loss of primary tooth substance. Primary teeth with coronal damage caused by trauma, caries and other problems require prosthetic or restorative treatments to restore function and aesthetics. Traditional stainless steel crowns (SSCs) have proven to be a very successful and durable material for restoring primary teeth with excessive substance loss [1].

Excessive material loss reduces the teeth’ resistance to functional force. SSCs are the greatest aid in restoring lost tooth structure. Currently, the main reason for using SSCs is to facilitate ideal chewing activity and vertical size continuity for a child. SSCs provide satisfactory adhesion and chewing function. SSCs are durable, i.e. they have long survival rates. In addition, no technical precision is required. Therefore, SSCs are commonly used for restorations in the posterior primary teeth [2, 3].

Dental cements are used for temporary and permanent restorations. They are used as a base material for pulp protection and for cementation of crowns. Several types of cements have been developed [4]. Non-adhesive cements (e.g. zinc oxide–eugenol, zinc phosphate, polycarboxylate, and zinc-reinforced copper cements) and adhesive cements (e.g. glass ionomer, resin-modified glass ionomer, and adhesive resin cements) are used to cement SSCs and zirconia pediatric crowns (ZPCs) [5, 6]. Each type of luting cement has some advantages and disadvantages. Dental cements are bonded to hard dental tissues and restorative and prosthetic materials by mechanical, chemical or physical mechanisms [7, 8]. Because of the differences in the properties of cement materials (adhesion, solubility, physical strength, and compatibility with hard tooth tissue), studies have focused on the adhesive strength, survival, and microleakage of luting cements used in the cementation of SSCs [5, 6, 9]. In general, it is desirable that luting cements do not stress the tooth, cause fracture under excessive pressure, or dissolve easily. They could also be easy to apply [9]. Although SSCs have a high clinical success rate, crown loss may occur due to cementation failure [10]. After cementation of SSCs, fractures, crushing or delamination may be observed in regions where stress is concentrated under functional force. This is due to the mechanical properties of the luting cements. Therefore, clinicians should consider the mechanical properties of luting cements when selecting materials for SSC cementation.

The type and thickness of material used to cement the crowns will affect the stress distribution on the restoration and on the teeth [11]. It has been reported that the cement used is particularly effective on stresses concentrated in the cervical region [12]. The use of glass ionomer cement for cementing SSCs and ZPCs has been reported in some clinical studies [5, 6, 13, 14]. The use of glass ionomer cement for SSCs and ZPCs has been reported in several clinical studies [5, 6, 13, 14]. These clinical studies evaluated various parameters such as retention, clinical success rate, number of fractured crowns and gingival index scores using SSCs and ZPCs cemented with glass ionomer cement [5, 6, 13, 14].

Resin cements require several application steps such as etching, priming and bonding. Recently, self-cure adhesive resin cements have been developed that eliminate the need for etching, priming and bonding as separate steps. Studies on SSC cemented with resin cement are limited [10, 15, 16]. These studies have reported the retention strength, microleakage, stress distribution patterns and deformations of SSC cemented with resin cement [10, 15, 16].

Finite element analysis (FEA) has been widely used and effectively applied in many fields of engineering, bioengineering and dentistry through numerical analysis. Clinical or experimental studies can be affected by many different factors such as variations in tooth anatomy, equipment calibration, author bias [17]. FEA is a valid method to address the mechanical performance as well as to interpret the mechanisms of experimental results [18, 19]. FEA helps researchers to obtain stress distributions of a complex structure under different scenarios, which is difficult to obtain from laboratory experiments [20–22]. FEA can focus on a particular factor, eliminating confounding issues that may arise in clinical practice or in the laboratory [20]. FEA is also less costly and time consuming than experimental research [23]. However, virtual FEA requires modelling and complex calculations with correct boundary conditions [24]. The stress distribution of teeth restored with SSC has been studied using FEA [10, 25–27]. However, the stress distribution of primary teeth restored with SSC using different luting cements has been studied by FEA to a limited extent [10, 25].

The purpose of this study was to evaluate the stress distributions of a primary molar tooth restored with SSC using resin and glass ionomer luting cements by FEA. The null hypothesis is that there will be no difference in the stress distributions of a primary molar tooth restored with SSC using resin and glass ionomer luting cements.

## Materials and methods

No tooth extraction was performed specifically for this study. In addition, no patient was asked to provide a computed tomography (CT) scan for this study. The decay-free, crack-free, orthodontically extracted primary molar was used to create a three-dimensional (3D) tooth model. This tooth was scanned by CT to obtain the original DICOM data. The original DICOM data were transferred to a computer program (Mimics 10.01, Materialise, Leuven, Belgium). A computer-aided design program (SolidWorks 2014 Premium, Concord, MA) was used to simplify the geometry and create a 3D model of the primary molar. The appropriate tooth preparation for SSC was simulated on a 3D solid model of the tooth. Modelling was performed with a 30 μm adhesive cement thickness.

**Fig. 1:**
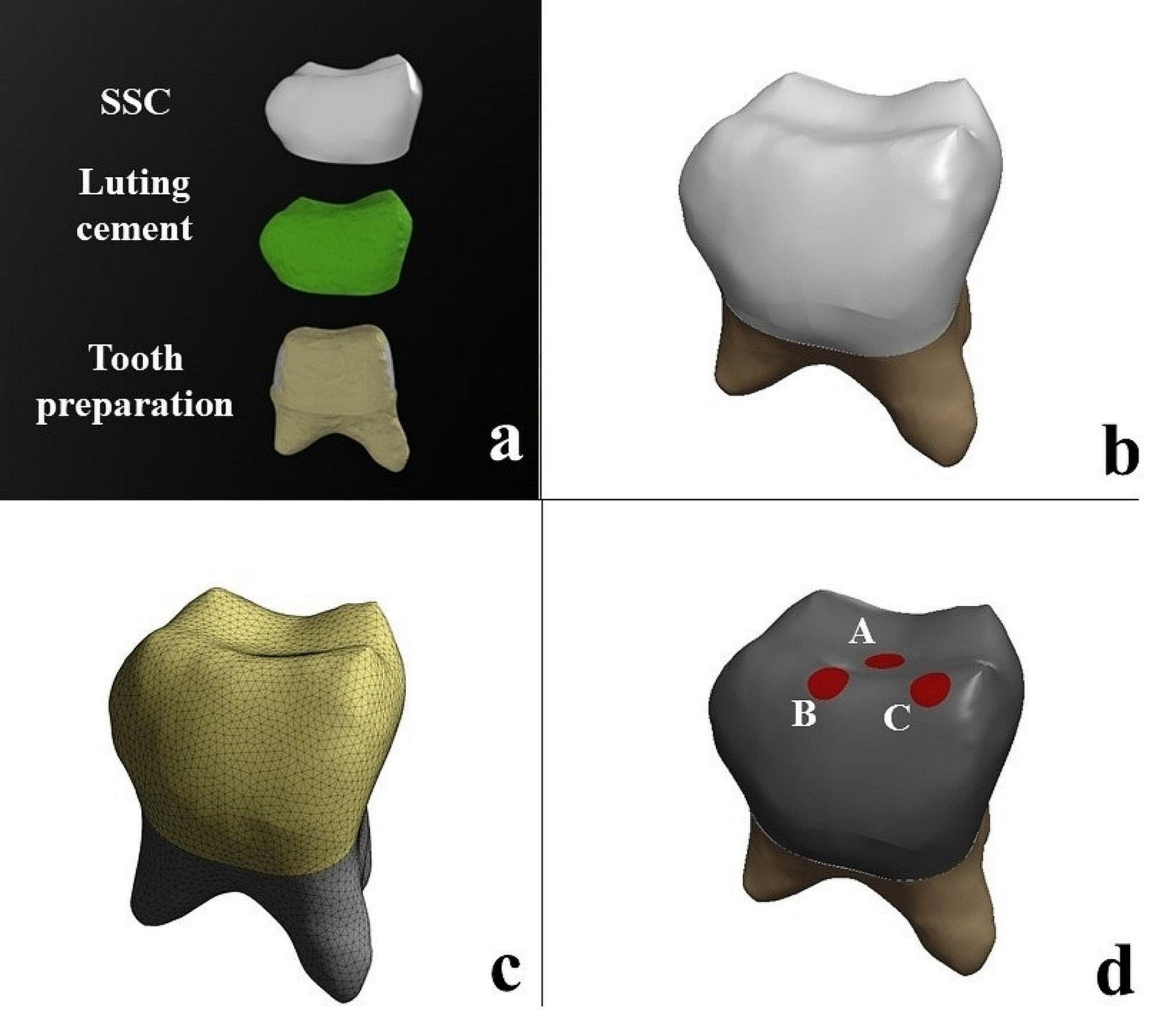
The preparation of 3D model. **a)** The simulation of tooth preparation for the SSC, **b)** The 3D model of SSC, **c)** The meshed model, **d)** The direction of force applications: vertical (Point A) and oblique (Points B and C)

In our study, only one tooth model was prepared as the tooth model with SSC (Fig. 1a and b). Two different luting cements were tested in this study: self-cure adhesive resin cement (RelyXUnicem Aplicap; 3 M ESPE, St Paul, MN, USA) and glass ionomer cement (KetacCem Maxicap; 3 M ESPE, St Paul, MN, USA). Stress analysis was performed on two numerical models according to the cement type. The models were transferred to ANSYS Workbench, version 13.0 (Swanson ANSYS Inc., Houston, PA, USA) for mathematical solutions and automatic mesh generation (Fig. 1c). In each model, 215,213 elements and 321,925 nodes were used.

All materials were assumed to be linear, homogeneous and isotropic. The material values (elastic modulus and Poisson’s ratio) defining the physical properties of each modeled structures were obtained from the manufacturer and published studies (Table 1) [26–30].

**Table 1:** Mechanical properties of the tooth and materials

Materials	Elastic modulus (GPa)	Poisson’s ratio
Enamel (primary teeth)	80.35	0.33
Dentine (primary teeth)	19.89	0.31
Stainless Steel Crown	200	0.33
Self-cure adhesive resin cement	4.9	0.27
Glass ionomer cement	20.1	0.3

The root of the tooth was selected as a fixed support in all dimensions (x, y, z) as a boundary condition. A previous study has shown that the bite forces in the primary dentition are in the range of 161-330 N [31]. Therefore, in the present study, a force of 330 N was applied to each model in vertical and oblique angulations to simulate the maximum occlusal and lateral chewing conditions [10, 31]. The forces were applied at three points (point A: vertical, point B and C: oblique) on the occlusal surface of the model (Fig. 1d). For the oblique loading, the force was applied from lingual to buccal, forming an angle of 45° with the long axis of the tooth. Static and linear analyses were performed. Maximum von Mises stress values were calculated in MPa units.

Stress distributions in the models are shown using colour scales. The values decrease from red to blue. Dark blue represents the areas of minimum von Mises stress and red represents the areas of maximum von Mises stress. The region with the highest stress value was defined as having the highest probability of failure and evaluations were made accordingly.

## Results

**Fig. 2:**
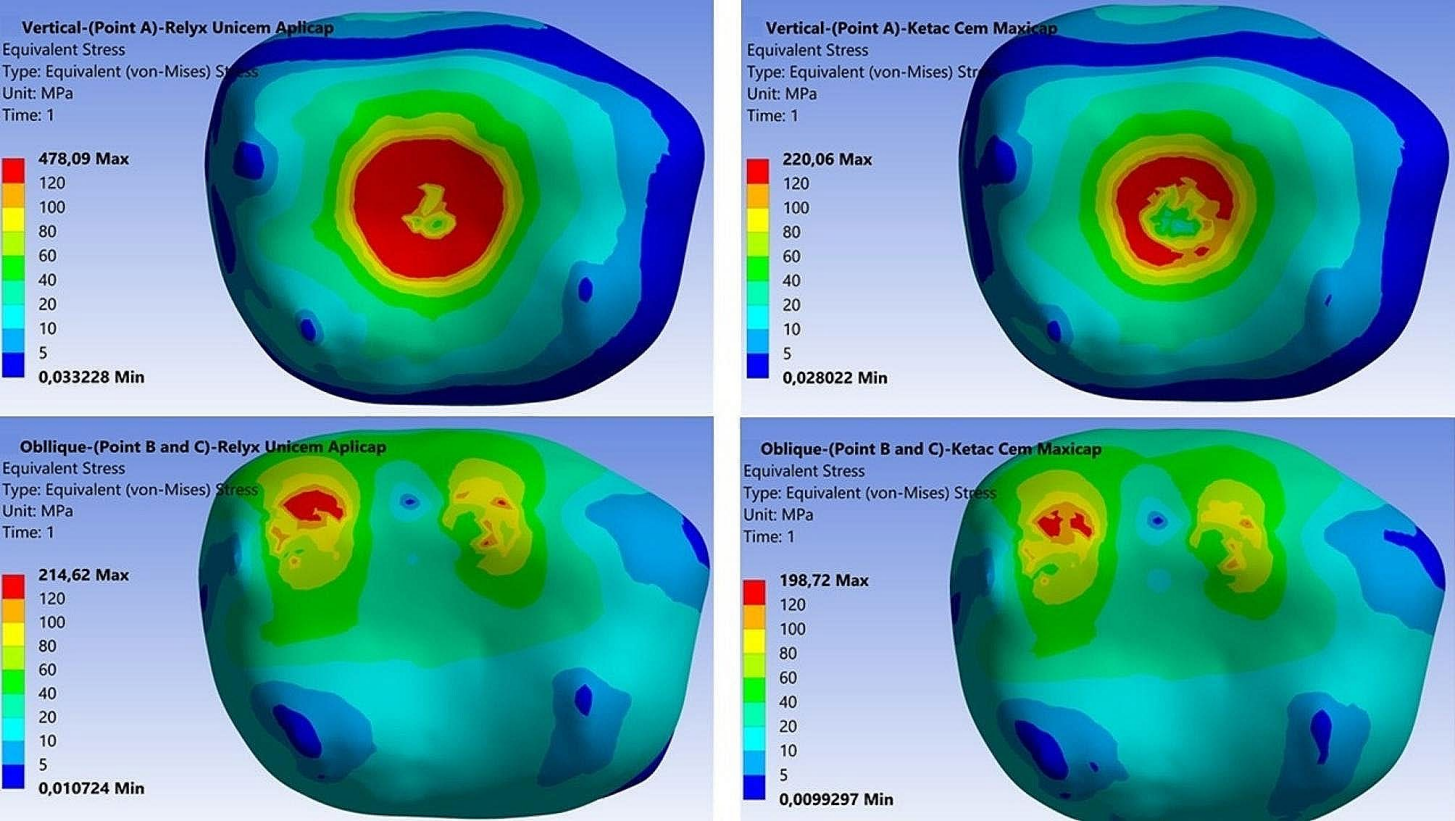
Von Mises stress patterns of models according to vertical and oblique loading on the occlusal plane

The maximum von Mises stress values of the models according to luting cement materials are presented in Table 2. When examining the stress values, the maximum von Mises stress value was observed in the area of force application for both vertical and oblique loading. In addition, the maximum von Mises stress values obtained for vertical loading were higher than those obtained for oblique loading. The maximum von Mises stress values were higher in the tooth model with SSC using self-cure adhesive resin cement (478.09 MPa and 214.62 MPa) than in the tooth model with SSC using glass ionomer cement (220.06 MPa and 198.72 MPa) for both vertical and oblique loading (Fig. 2).

**Table 2:** The maximum von Mises stress values of the models according to luting cement materials

Models	Load	The Von Mises Stress Values (MPa)
SSC using self-cure adhesive resin cement	Vertical	478.09
	Oblique	214.62
SSC using glass ionomer cement	Vertical	220.06
	Oblique	198.72

**Fig. 3:**
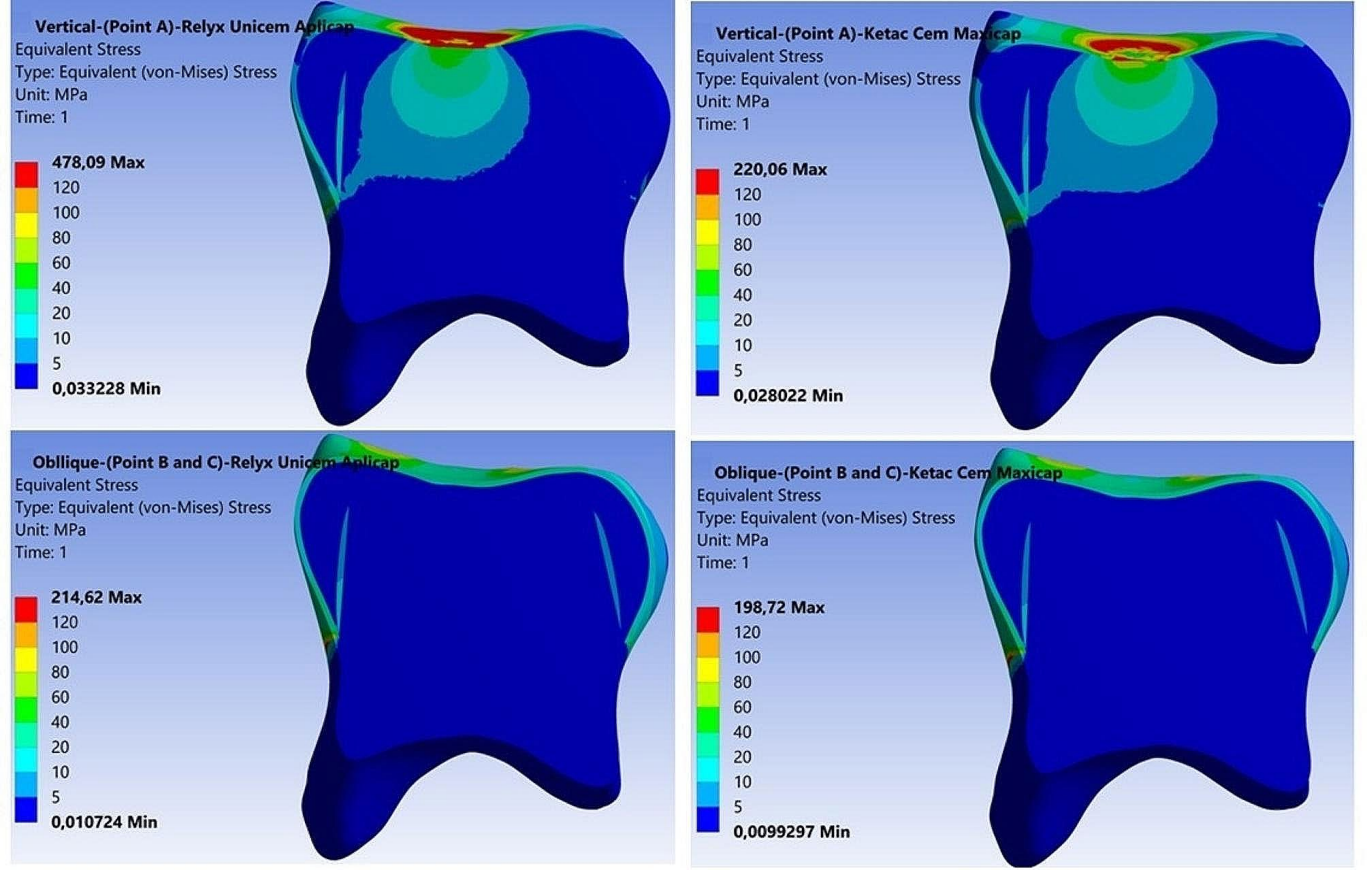
Von Mises stress patterns of models according to vertical and oblique loading on the cross-sectional plane

While the higher von Mises stress values were observed in the SSC, lower von Mises stress values were observed in the tooth tissues. The stress intensity accumulated at the occlusal contact points of the SSC progressively decreased towards the dentin layers due to the use of luting cement (Fig. 3).

## Discussion

Knowledge of the relationship between natural tissues and artificial units placed in the mouth is important for successful clinical treatment. The biomechanical study of the application of SSCs to primary molars presents some difficulties, including the 3D structure and several interacting variables. Several techniques have been used in clinics and laboratories to analyze the integrity of dental restorative materials under bite force. However, it is difficult provide a standard for the application of these methods in clinical studies in children due to ethical considerations. The use of the von Mises criterion in FEA facilitates the determination of the effects of force on restorations, converting normal and shear stress into a single stress and providing significant results [32]. The von Mises stress values have been used in FEA studies to evaluate the stress distributions resulting from the applied force [33–36]. Therefore, FEA and von Mises stress were used in the present study to better understand the biomechanical behavior of complex dental structures. The stress distributions of SSC cemented with self-cure adhesive resin and glass ionomer cements were investigated in primary molar and the null hypothesis was rejected according to the results of the present study.

Researchers have emphasized that the distribution of maximum von Mises stresses in FEA changes when the direction of the applied force and the area of application are changed [37, 38]. In biomechanical applications, the type of force selected and the way in which it is applied is controversial. Different values have been reported in the literature for maximum bite forces in the primary dentition. Owais et al. [39] reported 176 N as the maximum bite force in early primary dentition, whereas Abu Alhaija et al. [40] reported 197 N. Rentes evaluated the bite force according to the type of occlusion (normal occlusion, open bite and cross bite) in a group of children with primary dentition and found that the values ranged from 161 to 330 N [31]. When examining the studies evaluating the stress distribution of stainless steel crowns in primary teeth, Prabhakar et al. [26, 27] used the maximum bite force as 245 N by averaging the range of 161–330 N, while Waly et al. [10] evaluated using 330 N. In accordance with the studies made in our study, 330 N load was applied to simulate the maximum bite force. In the present study, normal occlusion was simulated and different malocclusions or force levels were not evaluated.

Rekow and Thompson reported that cement thickness can vary between 20 and 200 μm [41]. Sagsoz and Yanikoglu stated that the effect of cement thickness determined as 30, 90 and 150 μm, on fracture resistance was found to be similar [42]. In accordance with this literature, a cement thickness of 30 μm was preferred in our study to simulate minimally invasive preparation and to provide a standard for cement thickness.

Authors have reported that materials with high elastic modulus have greater durability and less deformation than materials with lower elastic modulus under the same degree of load [43–45]. These results are consistent with the results of the present study. Considering the results of the present study, the lower von Mises stress values were obtained in the SSC model with glass ionomer cement which has the higher elastic modulus. According to the results of our study, it can be said that the elastic modulus of the luting cement plays an important role in the maximum von Mises stress values obtained in the SSC. As the elastic modulus of the luting cement increased, the stress developed against the occlusal force decreased. Depending on the magnitude of the occlusal force on the SSC, fracture of the luting cement materials could occur if the stresses exceeded the endurance limit of the luting cement. In addition, it is important to balance the occlusal contact areas to maintain bond between the restorative material and tooth structure to prevent unwanted bite forces.

There have been limited studies on the stress distribution in SSCs according to cement type [10, 25]. Waly et al. showed that the distribution patterns of resulting stresses and deformations did not change with the type of cement (zinc phosphate, glass ionomer, resin-modified glass ionomer and resin), while the values were altered [10]. This result is not compatible with the results of our study and the difference may be due to methodological differences applied in the studies, such as different cement thickness and loading. Guduk et al. reported that the use of ZPC as a crown (ZPC and SSC) and glass ionomer cement as an adhesive material (glass ionomer, resin-modified glass ionomer and resin) in endodontically treated teeth reduces the possibility of fracture due to the stress generated during biting in dentin and crown [25]. This finding is consistent with the results of our study.

There are some limitations to this study. First, only two luting cements were tested, so results may vary if different cements are used. Second, the mandibular primary second molar was used for testing; results may vary if different teeth are used. Third, normal occlusion was simulated in our study; results may vary if different malocclusions or force levels are used. Fourth, no periodontal ligament or jaw modelling was performed in this study. In future studies, these models could be used to evaluate the loads distributed to the jaw. Finally, the results may vary with different parameters such as luting cement thickness or elasticity, malocclusion, isotropy, bite angle or forces, and other failure criteria such as principal stress may also be considered. Further research should be conducted to evaluate the effect of different parameters on the stress distribution in SSC for primary molars.

## Conclusion

Within the limitations of this FEA study, the following conclusions can be drawn:

1. The maximum von Mises stress values were higher in the tooth model with SSC using self-cure adhesive resin cement (478.09 MPa and 214.62 MPa) than in the tooth model with SSC using glass ionomer cement (220.06 MPa and 198.72 MPa), both for vertical and oblique loading, respectively.
2. In the restoration with SSC of children’s primary molars, cementation with glass ionomer cement can help to reduce the maximum von Mises stress values.

## Data Availability

Personal data of the participant will not be shared. The data used and/or analyzed during the current study are available from the corresponding author on reasonable request.
